# Prevalence of perceived stress and associations to symptoms of exhaustion, depression and anxiety in a working age population seeking primary care - an observational study

**DOI:** 10.1186/s12875-015-0252-7

**Published:** 2015-03-19

**Authors:** Lilian Wiegner, Dominique Hange, Cecilia Björkelund, Gunnar Ahlborg

**Affiliations:** Institute of Medicine, Department of Public Health and Community Medicine, The Sahlgrenska Academy at Gothenburg University, Box 454, SE-40530 Gothenburg, Sweden; Institute of Stress Medicine (ISM), Carl Skottbergs gata 22 B, SE- 413 19 Gothenburg, Sweden

**Keywords:** Primary health care, Stress, Burnout, Exhaustion disorder, Depression, Anxiety, Working age

## Abstract

**Background:**

Prolonged stress may lead to mental illness, but the prevalence of stress in a working age population seeking primary health care for whatever reason, is unknown. This paper seeks to examine to what extent this group perceives stress, as well as symptoms of burnout/exhaustion, depression and anxiety.

**Methods:**

In 2009, 587 primary health care patients aged 18–65 years (377 women, 210 men), with an appointment with a primary health care physician, participated in the study.

A screening questionnaire with questions about age, gender, marital status, employment, reason for medical consultation, and the QPS Nordic screening question about stress was distributed:” Stress is defined as a condition where you feel tense, restless, anxious or worried or cannot sleep at night because you think of problems all the time. Do you feel that kind of stress these days? There were five possible answers; “not at all” and ”only a little” (level 1),“to some extent” (level 2),“rather much” and “very much” (level 3). In a second step, symptoms of burnout/exhaustion (Shirom-Melamed Burnout Questionnaire and the Self-rated Exhaustion Disorder instrument) and anxiety/depression (Hospital Depression and Anxiety scale) were assessed among those with higher levels of perceived stress.

**Results:**

345 (59%) of the study patients indicated stress levels 2 or 3 (237 women and 108 men). Women more often indicated increased levels of stress than men. Two thirds of the participants expressing stress levels 2–3 indicated a high degree of burnout, and approximately half of them indicated Exhaustion Disorder (ED). Among highly stressed patients (level 3), 33% reported symptoms indicating possible depression and 64% possible anxiety.

**Conclusion:**

More than half of this working age population perceived more than a little stress, as defined, women to a greater extent than men. Symptoms of burnout and exhaustion were common. A high level of perceived stress was often accompanied by symptoms of depression and/or anxiety.

**Electronic supplementary material:**

The online version of this article (doi:10.1186/s12875-015-0252-7) contains supplementary material, which is available to authorized users.

## Background

Stress-related illness is believed to be one reason for the increase in long-term sick leave during the last decade in many European countries [1-3]. Over the last ten years, work-related stress has increased in nine EU countries [4]; 80% of the general population in European countries believe that work related stress will increase in the coming five years [5]. In 2009, stress was measured in a Swedish population aged 16–84, using the General Health Questionnaire (GHQ 12); 13% reported stress and 3% reported severe stress [6]. Work-related stress is relatively well studied, and there are several valid theoretical models available [7,8]. High mental demands and increasing workloads, as well as psychological injustice are some factors connected to work related stress [1].

One consequence of chronic stress is burnout [9], a related condition is Exhaustion Disorder (ED). The clinical diagnosis ED has been proposed by the National Board of Health and Welfare in 2005, to improve diagnostics in case of stress-related exhaustion and was assigned the code F43,8A of the international Classification of Diseases and related Health Problems (ICD-10) [10] (Additional file 1: Diagnostic criteria for Exhaustion disorder). The diagnostic criteria includes exhaustion, cognitive dysfunction, sleep problems and somatic symptoms. Its dominating feature is lack of energy [11]. The diagnostic procedure for ED has previously been described in detail [12]. ED defines patients with exhaustion that has developed as a consequence of identifiable stressor(s) that have been present for at least six months and is defined by clinical criteria, including somatic, as well as mental health symptoms that can arise as a consequence of all sorts of perceived stress [10]. Burnout is a psychological concept, but lacks defined diagnostic criteria [13]. We have previously shown that the majority of patients fulfilling criteria for ED fulfilling the burnout criteria as well [11]. The ED diagnosis has so far been in formal use only in Sweden. There are few studies of ED in different Swedish populations [11,14], but, to our knowledge, no waiting room studies of the relationship between perceived stress and ED in primary care populations.

Depression and anxiety are common mental disorders, well studied in various populations. In epidemiologic studies from different countries, 4-10% of the participants aged 20–60 years were deemed to suffer from depression [15], and 12-17% of the population suffered from various anxiety syndromes [16]. The point prevalence of major depression in Sweden was 5,2% and the point prevalence of clinical significant anxiety was 14,7% in an general population 2009 [17]. This is in line with 12 month prevalence rates of depression from US (6,7%) [18], Australia (6,3%) [19] and the Netherlands (5,8%) [20]. Previous studies from Germany showed a point prevalence of anxiety 12,1% [21] and from US 18,1% [18]. The prevalence of Burnout has been studied in Sweden and high burnout (above 4,0 on SMBQ) was reported by 13% of the population studied [22] General practitioners (GPs) play a central role when it comes to diagnosing and treating of common mental disorders, since consultations in cases of depression and anxiety mostly take place in primary care in Sweden [23], as well as in other countries [24]. In 2009, Hanel et al. showed that patients diagnosed by their GPs with depression or anxiety were often also suffering from psychosocial and/or financial stress [25]. Co-morbid symptoms of depression and/or anxiety are commonly seen in clinical practice among patients diagnosed with ED [11]. The prevalence of perceived stress in a working age population seeking primary care is still unknown, as well as the prevalence of symptoms of depression and anxiety in the group perceiving stress. The aim of this study was to assess the extent to which people of working age, seeking primary care physicians, experience stress, and if there were differences regarding gender, age, marital status and employment status between the stress level groups. In a second step we wanted to assess symptoms of burnout, exhaustion, anxiety and depression among those reporting higher levels of perceived stress.

### Study population

The study population consisted of primary care patients aged 18–65 years who had an appointment with a primary care physician at any one of five participating health centres in the Gothenburg area, during 2–5 consecutive days in the spring and autumn respectively, of 2009. The five centres were recruited by an open invitation to health centres in the Gothenburg area, and selected to ensure socio-economic representativeness. They were publicly financed and geographically spread, and included inner city, suburban and small town residents. The number of working GPs at each health centre varied from four (suburban) to 13 (inner city), and the total amount of listed patients varied from around 4,200 to 15,000.

The appointments were pre-booked, but in cases of acute disease or exacerbation of chronic disease, they could have been booked on the same day as the contact was made. Most of the acute appointments were made by telephone, following advice from a nurse. Everybody in the selected age group was asked to participate in the study, regardless of reason for the doctor’s appointment. Of 689 eligible patients, 587 agreed to participate (85%). The most common reason to decline participation was lack of interest or time. To avoid bias due to seasonal variation in mental health (depression), half of the study participants at each health centre were included during spring, and the other half during the autumn. All persons included gave written, informed consent. The study was approved by the Regional Ethical Review Board in Gothenburg, Sweden.

## Methods

Regular health centre staff distributed a screening form (screening step 1) “Investigation of Stress among primary care patients”, shown in Additional file 2. The screening form contained questions about age, gender, marital status, employment status and stress and was distributed to the participants while they were while waiting for their doctor’s appointment. The screening questionnaire was developed for this study. It was first tested in a feasibility study to clarity and face validity. The specific stress question from QPS Nordic used within the questionnaire, was a previous validated (described below). A question about the cause (type of health problem) of the visit was included. Age was divided into three age groups; 18–24 years, 25–39 years and 40–65 years, based on both a social and a working life perspective. The marital status options were: married/cohabiting, single, or living with parents. Occupational status options were: employed, student, unemployed, or other. The “other”-alternative was followed by a request to specify in writing.

Data collection was performed in three steps. First, all participants filled in the screening step 1 questionnaire. In a second step those who gave answers indicating increased stress were asked to fill in three multi item self-report scales, measuring symptoms of exhaustion, burnout, depression and anxiety. The third step was a doctors appointment with a specialist from specialist stress centre (not reported in this article).

### Measure of stress

We used a validated, single stress question from The Nordic Questionnaire for Psychological and Social Factors at Work (QPS Nordic) in order to assess perceived stress. This measure was shown to have satisfactory content, criterion and constructed validity [26] and seemed feasible to use for the initial screening. The stress definition and question reads as follows: “Stress means a situation in which a person feels tense, restless, nervous or anxious, or is unable to sleep at night because his/her mind is troubled all the time. Do you feel this kind of stress these days?” There were five possible answers: “not at all”, “only a little”, “to some extent”, “rather much” and “very much”. The answers “ not at all ” and “only a little” were labelled very low levels of perceived stress (level 1), and “to some extent” was labelled a low level of perceived stress (level 2) while “rather much” and “very much”, were labelled moderate to high levels of perceived stress (level 3). Those scoring self-rated stress level 2 or 3 were asked to continue to the second step of the investigation, in which they completed the self-rating scales regarding symptoms of burnout, exhaustion, depression and anxiety.

### Burnout and stress-related exhaustion

The Shirom-Melamed Burnout Questionnaire (SMBQ), was used to measure symptoms of burnout [13]. The scale consists of 22 statements, and the responses are recorded on a seven-point Likert scale varying from ‘almost never’ to ‘almost always’. SMBQ contains four subscales: ‘burnout’, ‘tension’, ‘listlessness’ and ‘cognitive weariness’. The total mean score ranges from one to seven. Overall, the SMBQ questionnaire correlates highly with the emotional exhaustion subscale in the Maslach Burnout Inventory [27]. A total mean score of 3.75 was used as the cut-off point for increased symptoms of burnout. This choice was made based on earlier observations [27].

An instrument designed for the assessment of self-rated Exhaustion Disorder (s-ED) designed by the research group at the Institute of Stress Medicine (ISM) and based on the diagnostic criteria for ED [14], was used to identify participants with symptoms of stress-related exhaustion. This instrument was designed to promote sensitivity rather than specificity, and has shown good construct and predictive validity in a longitudinal study of health care and social security employees [14]. It includes four items concerning physical and mental exhaustion, long-term stress exposure, symptoms and their accompanying degree of suffering/functional impairment [14]. Item four reads: “Have the complaints above (question 1–3) markedly decreased your well-being and/or your functional capacity (work ability, family life, leisure activities or other important areas?)”. The response alternatives were “No”, “Yes, somewhat” and “Yes, pronounced”, and the latter two were collapsed into one category “possible ED”.

### Depression and anxiety

Finally, the Hospital Anxiety and Depression scale (HAD), originally developed for non-psychiatric clinics to detect states of depression and anxiety [28], was used to assess self-reported symptoms of depression and anxiety. The scale consists of 14 statements; seven for each of the two subscales. The participants chose one of four responses for each statement concerning mood changes that may occur during the course of depression or anxiety. Both subscales use the sum scores. HAD has been found to have satisfactory reliability and validity [29], and has also been shown to be sensitive in reflecting changes over time, in response to different interventions. A score of > 7 was, in this study, used to define symptoms indicating possible or probable depression and anxiety, respectively.

### Statistics

Analyses were carried out in SPSS version 20.0. Results from the screening for stress (step 1), burnout, s-ED, depression and anxiety (step 2) are presented as numbers (n) and proportions (%) in each category. Differences were tested for statistical significance by Chi-Square test. The level of significance was set at p < 0.05.

## Results

### Patients’ characteristics

A total of 587 persons answered the screening questionnaire, 377 (64%) women and 210 (36%) men. Of these, 242 (41%) scored perceived stress level 1 (women 37%, men 49%), 153 (26%) stress level 2 (women 26%, men 26%), and 192 (33%) stress level 3 (women 36%, men 26%). Mean age was 42 years (range 18–65). There were differences between the stress level groups regarding gender and marital status i.e. the proportion of women were higher and the proportion of singles increased with increasing level of stress (Table 1). Most of the participants were employed. Eighty-eight participants chose the alternative “other” regarding employment, and 67 of these specified this as being on parental leave, sick leave, having disability pension or early retirement, or being self-employed. A higher proportion of participants in the 40–65 age group (18%) chose this alternative, most of them because they were retired. Among participants in the lowest stress level, a high proportion reported being on parental leave (32%).

**Table 1:** **Characteristics of participating primary care patients reporting different stress levels,** ^**1**^ **and of the total population**

**Study variable**	**Level 1**		**Level 2**		**Level 3**		**Total**	
	**n = 242**		**n = 153**		**n = 192**		**n = 587**	
	**n**	**%**	**n**	***%***	**n**	***%***	**n**	***%***
**Gender**								
Women	140	*58*	99	*65*	138	*72*	377	*64*
Men	102	*42*	54	*35*	54	*28*	210	*36*
**Age**								
18-24 years	26	*11*	20	*13*	25	*13*	71	*12*
25-39 years	*80*	*33*	51	*33*	70	*36*	201	*34*
40-65 years	136	*56*	82	*54*	97	*51*	315	*54*
**Marital status**								
Married/cohabiting	179	*74*	103	*67*	110	*58*	392	*67*
Single	60	*25*	47	*31*	79	*41*	186	*32*
Living with parents	2	*1*	3	*2*	2	*1*	7	*1*
**Employment**								
Employed	179	*74*	107	*70*	121	*63*	407	*69*
Student	21	*9*	17	*11*	25	*13*	63	*11*
Unemployed	6	*2*	6	*4*	17	*9*	29	*5*
Other^2^	36	*15*	23	*15*	29	*15*	88	*15*

^1^Levels of stress were measured using the following question from the QPS Nordic instrument: “Stress means a situation in which a person feels tense, restless, nervous or anxious or is unable to sleep at night because his/her mind is constantly troubled. Do you feel this kind of stress these days?” “Not at all” and “only a little = level 1, “to some extent” = level 2, “rather much” and “very much” = level 3.

^2^On parental leave, disability pension, sick leave, early retirement or being self-employed.

The 345 patients (59%) who indicated that they suffered from more than just a little stress (level 2 or 3) were asked to participate in the second part of the study, in which symptoms of burnout, exhaustion, depression and anxiety were assessed. In total, 305 patients (216 women and 89 men) with a mean age of 41 years (range 18–65) agreed to participate in this part of the study. This subgroup was similar to the total study population regarding age distribution and marital status; 53% (n = 162) were between 40 and 65 years old, and 62% were married or cohabiting.

### Symptoms of burnout, exhaustion, depression and anxiety

Complete answers to the self-rating scales were obtained from 92% for SMBQ, 96% for s-ED, 94% for HAD-depression, and 93% for HAD-anxiety. Seventy-two percent of the respondents scored above the cut-off point for symptoms of burnout, 57% scored possible ED, 35% scored above the cut-off point for symptoms of depression, and 71% for symptoms of anxiety. Fifty-four percent of the respondents met our criteria for both burnout and s-ED.

**Figure 1:**
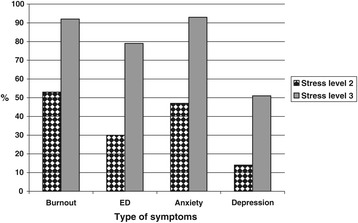
**Differences among female participants in proportion (%), between perceived stress level 2 (n = 86) and stress level 3 (n = 130), regarding symptoms of burnout, exhaustion, depression, and anxiety.** (p < 0.01 for all differences). Burnout = Shirom-Melamed Burnout Questionnaire mean total score > 3,75; ED = Self-rated Exhaustion Disorder; Anxiety and Depression = Hospital Anxiety and Depression subscale score >7.

**Figure 2:**
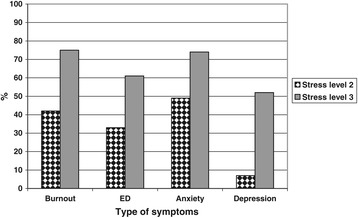
**Differences among male participants in proportion (%), between perceived stress level 2 (n = 86) and stress level 3 (n = 130), regarding symptoms of burnout, exhaustion, depression, and anxiety.** (p < 0.01 for all differences). Burnout = Shirom-Melamed Burnout Questionnaire mean total score > 3,75; ED = Self-rated Exhaustion Disorder; Anxiety and Depression = Hospital Anxiety and Depression subscale score >7.

In the stress level 2 group about half (49%) scored above the cut-off point for symptoms of burnout, and a little more than one third (32%) gave responses indicating possible ED. In the group with the highest level of stress 87% indicated burnout and 74% possible ED. The proportion of participants with symptoms of burnout and/or ED was larger among those who reported stress level 3, compared to those reporting stress level 2 for both women (p < 0,05) and men (p < 0,05) (Figures 1 and 2).Symptoms of burnout and ED as well as anxiety were significant more common among women than among men, at stress level 3 (p < 0,05)

.

Depressive symptoms were more common among those reporting most stress, 51% compared to 12% at stress level 2 (p < 0,05). There were small differences between men and women in this respect within each stress level group. Symptoms of anxiety were more common, and more than nine out of ten women in the group reporting most stress experienced such symptoms.

To explore to what extent symptoms of burnout or exhaustion coexisted with symptoms of depression or anxiety, the 272 patients reporting stress levels 2 or 3, who had completed all four self-rating scales, were identified. Sixty-eight percent scored above cut-off within both of these two categories of mental symptoms, while 15% reported neither (p < 0,01) (Table 2) At stress level 2, 40% indicated both categories while 86% at stress level 3 reported both categories (p < 0,01).

**Table 2:** **Distribution of participants with self-rated symptoms of burnout/exhaustion and/or depression/anxiety (D/A), within stress levels two and three respectively**

	**Stress level 2**				**Stress level 3**				**Total**			
	**n = 108**				**n = 164**				**n = 272**			
	**Symptoms of D** ^**3**^ **/A** ^**4**^				**Symptoms of D** ^**3**^ **/A** ^**4**^				**Symptoms of D** ^**3**^ **/A** ^**4**^			
Symptoms of burnout^1^/exhaustion^2^	No		Yes		No		Yes		No		Yes	
	***%***	n	***%***	n	***%***	n	***%***	n	***%***	n	***%***	n
No	*31*	34	*10*	11	*5*	8	*4*	7	***15*** ^*^	42	*7*	18
Yes	*18*	20	40**	43	5	8	86**	141	*10*	28	***68*** ^*^	184
Total within each stress level	*50*	54	*50*	54	*9*	16	*91*	148	*26*	70	*74*	202

*Difference between patients in the total study population reporting symptoms of depression/anxiety and coexisting burnout/depression and patients reporting neither of these symptoms.(p<0,01).

**Difference between proportion reporting symptoms of depression/anxiety and coexisting symptoms of burnout/exhaustion between stress level 2 (40%) and stress level 3 (86%). (p=<0.01).

^1^Shirom-Melamed Burnout Questionnaire (SMBQ) Mean total score ≥ 3,75.

^2^Self rated Exhaustion Disorder (s-ED) Moderate to severe score.

^3^Hospital Depression scale (HAD-D) >7.

^4^Hospital Anxiety scale (HAD-A) >7.

## Discussion

### Perceived stress

Using the QPS Nordic single item definition, more than half of the primary care population in this study reported some stress, women generally more often than men. One third of women perceived rather much/very much stress, but only about one of four men. The same measure of stress was used in a longitudinal study of more than 3000 employees (87% women) in public health care and social insurance offices in the same geographical region. In that population, the prevalence of rather much/very much stress was 17% at baseline in 2004 [30]. It seems reasonable that the prevalence of high levels of stress among persons of working age seeking primary care is about double that of employees in this female dominated sector of the labour market [6]. Earlier studies have indicated that women’s domestic work takes more time than men’s [31,32]. This could be one plausible explanation, at least to some extent, to why women experience higher levels of stress than men.

There was no indication that experience of stress was related to age, i.e. there was a similar age distribution in the three different stress level groups. A Finnish study from 2003 among employees 25–64 years of age, in which the single stress item was validated, indicated that the stress symptoms increased with age [26]. The lack of such an effect of age in our study population could possibly be explained, at least partly, by the fact that all participants experienced a health problem, a stress exposure in itself.

A higher proportion of singles was found in the high stress level group. We did not separate singles living with or without children, however. In a Scandinavian study, working single mothers reported higher stress levels than working non-single mothers [33]. A similar finding was made in a study by Kushnir et al. [33,34]. Low income, common among single mothers, was associated with higher psychosocial stress in a recent study of 3000 single mothers in Germany aged 17–60 years [35]. Married women on the other hand, are more likely to report somatic discomfort according to a study by Nakao et al. [36]. It is tempting to assume that single mothers were overrepresented among the highest stress level group.

There was a low proportion (5%) of unemployed persons in our study population. Nevertheless, there was an indication that unemployed people were overrepresented in the higher stress level group. Unemployment, as well as low income, was associated with a higher prevalence of psychological distress in a study of hospitalized patients in Israel [37]. This was confirmed in a Japanese study [38]. It was interesting to note that a rather high proportion of our participants in the lower stress category reported being on parental leave (32%). The Swedish social insurance system makes it possible to be on parental leave with economic compensation for a long time compared to most other countries, which might contribute to a lower level of stress during this period compared to being a working parent.

### Symptoms of burnout, ED, depression and anxiety

Primary care patients, especially women, who perceive stress, seem to experience symptoms of burnout as well as ED to a greater extent than what we would expect in a general population sample with the same gender and age distribution. Recent Swedish general population surveys, using various burnout measures [22,39], have indicated a high prevalence of burnout; between 13% and 21%. The Swedish study among healthcare workers and social insurance officers by Glise et al.(2004), using the same measure as this study [14] showed a prevalence of burnout of 26%. The same study showed a prevalence of ED of 16%; less than one third of the prevalence in our population of patients seeking primary care. Self-reported ED was more common among women than men, especially in the highest stress level group of our study. A perception of s-ED does not mean that the respondent actually has ED, but rather reports symptoms indicating increased risk of developing or possibly having this condition. A high SMBQ score can be interpreted in a similar way in relation to clinical burnout.

Discriminant validity between burnout and major depression has been studied [9], and both conditions share common features such as cognitive weariness, but they are not identical. A major dissimilarity is a feeling of loss of status and “giving up” which is often reported by patients with depression [40]. We were interested to find out to what extent patients in a primary care setting, who score high on measures of burnout and exhaustion, also show co-morbid symptoms of depression and/or anxiety. A previous waiting room study in Sweden (n = 1392) indicated that 17% had symptoms of depression (HAD-D >7) and 29% had symptoms of anxiety (HAD- A >7) regardless of stress [41]. No gender differences were found. The population was similar to ours in terms of gender proportion (38% men and 62% women), but also included patients over 65 years. We did not assess symptoms of depression or anxiety among all participants, but among those reporting stress the prevalence was high, 35% and 71% respectively. The symptom scores thus increased with the level of stress. It is well known from previous studies that in a general population, depression is about twice as common among women as among men [15]. In our study, in the group with a low level of stress, depression was twice as common among women, while, surprisingly, no gender difference was seen in the group reporting pronounced stress.

Both men and women with stress symptoms suffered from a high degree of anxiety symptoms, especially those reporting the highest stress level.

It is previously known that depression and anxiety with co-morbid ED is associated with a long recovery period [11]. Among patients with symptoms of depression/anxiety, it is thus essential to identify significant stressor exposure and possible co-morbid ED.

### Methodological considerations

Screening for stress with a single item may seem too simple to capture such a complex concept. We decided to use this method for two main reasons: feasibility and validity. It was obvious that an extensive questionnaire or interview could not be used in the first screening step of all patients with a doctor’s appointment in the waiting rooms. The single item used was previously validated and shown to correlate well with e.g. established burnout scales [26]. There are several instruments available to assess burnout, depression and anxiety. We have previously used both SMBQ and HAD in studies of working populations as well as in clinical studies [11,14]. The instruments have also been validated in such populations [42].

### Strengths and limitations

The study had a high response rate (85%). This was partly due to the use of an easily understandable questionnaire, distributed by regular staff at the five participating health centers. These health centers represented both urban and more rural districts, to avoid selection on socio-economic grounds. We believe the sample in the study to be fairly representative of patients in a primary care population, at least in similar Nordic contexts.

All instruments used were at least partly validated, and the QPS Nordic screening question regarding stress used in the screening form, included a definition of stress which was not dependent on the source of the stress (work or home). To be useful in a primary care setting, it was important that the instrument used referred to the general experience of stress in a working population, in a Nordic context. In the first two steps of this study, we were not able to distinguish between different sources of stress, however.

There could be a restriction in the definition of stress in the screening item, since it includes anxiety symptoms and sleep disturbance, but not, e.g., stress-related somatic symptoms. Words like anxiety and nervousness are likely to be considered negatively charged words, especially by men of a younger age. This could result in an underestimation of stress experience.

The study results cannot be extrapolated to other countries as it come to ED, since this is a diagnose not used in other countries yet.

It may be seen as a limitation that the assessments of stress and burnout/exhaustion, as well as symptoms of anxiety and depression, were dependent on self-reports. Numerous studies reveal that women are more likely to self-report health-related problems than men [43]. Men also seek medical care to a lower extent than women, and this should be taken into consideration when comparing the results with those from e.g. general population studies.

More knowledge is needed in order to better understand and handle the increasing problem of mental disorders among patients seeking primary care. This study contributes to existing knowledge by giving an indication of the extent to which primary care patients perceive stress and repot symptoms of burnout, exhaustion, depression and anxiety. Our intension further on is to find out whether there are any special reasons for encounters that correlate with diagnose ED with or without co-existing depression and/or anxiety. By analysing data from the clinical specialist examination (step three) hopefully we will find methods for early identifying patients with or at risk of developing ED, and thereby likely prevent long lasting sick leave.

## Conclusions

Our study indicates that a working age population seeking primary health care (for whatever symptoms) perceive high levels of stress and symptoms of ED, depression and anxiety to a much higher extent than a general population. More than half of this primary care population of working age perceived more than a little stress. Two thirds of the participants expressing such stress, indicated a high degree of burnout, and approximately half of them possible ED. Previous studies have shown that women, singles and unemployed people perceive stress to a higher extent, which could be confirmed in this primary care population as well. Reporting symptoms indicating probable depression and/or anxiety was more common in the group reporting higher stress levels.

## Additional files

Additional file 1:
**Diagnostic criteria for Exhaustion disorder.**
Additional file 2:
**Screening form “Investigation of stress among primary care patientens”.**
