# Supplementary material for: Prevalence of perceived stress and associations to symptoms of exhaustion, depression and anxiety in a working age population seeking primary care - an observational study
Source: BMC Fam Pract. 2015 Mar 19;16:38. doi: 10.1186/s12875-015-0252-7 (PMC4377029; doi:10.1186/s12875-015-0252-7)
Supplement: Additional file 1: — Diagnostic criteria for Exhaustion disorder. [file 12875_2015_252_MOESM1_ESM.docx]

**Diagnostic criteria for stress-related Exhaustion disorder as proposed by the Swedish National Board of Health and Welfare 2005**

A Physical and mental symptoms of exhaustion with minimum two weeks duration. The symptoms have developed in response to one or more identifiable stressors which have been present for at least 6 months.

B Markedly reduced mental energy, which is manifested by reduced initiative, lack of endurance, or increase of time needed for recovery after mental efforts.

C At least four of the following symptoms have been present most of the day, nearly every day, during the same 2 week period:

1 Persistent complaints of impaired memory.

2 Markedly reduced capacity to tolerate demands or to work under time pressure.

3 Emotional instability or irritability.

4 Insomnia or hypersomnia.

5 Persistent complaints of physical weakness or fatigue.

6 Physical symptoms such as muscular pain, chest pain, palpitations, gastrointestinal problems, vertigo or increased sensitivity to sounds.

D The symptoms cause clinically significant distress or impairment in social, occupational or other important areas of functioning.

E The symptoms are not due to the direct physiological effects of a substance (e.g. a drug of abuse, a medication) or a general medical condition (e.g. hypothyroidism, diabetes, infectious disease).

F The stress-related disorder does not meet the criteria for major depressive disorder, dysthymic disorder or generalized anxiety disorder.
