# Supplementary material for: Prevalence of perceived stress and associations to symptoms of exhaustion, depression and anxiety in a working age population seeking primary care - an observational study
Source: BMC Fam Pract. 2015 Mar 19;16:38. doi: 10.1186/s12875-015-0252-7 (PMC4377029; doi:10.1186/s12875-015-0252-7)
Supplement: Additional file 2: — Screening form “Investigation of stress among primary care patientens”. [file 12875_2015_252_MOESM2_ESM.doc]

Date:………………………..……………..

Name:…………………………………….…

ID number:…………………………..…

# Investigation of Stress among primary care patients

Age ………… ❑ Woman ❑ Married/Cohabiting

❑ Man ❑ Single

Occupational status:

❑ Employed 🡪 Do you have access to occupational health?  Yes

❑ Student  No

❑ Unemployed  Don’t know

❑ Other……………………………..

Why do you seek medical care today?

(e.g due to stomach pain, headache, infection, control of blood pressure, if several symptoms please specify all)

………………………………………………………………………………………………………………………….

Stress means a situation in which a person feels tense, restless, nervous or anxious, or is unable to sleep at night because his/her mind is troubled all the time. Do you feel this kind of stress these days?

❑ Not at all

❑ Only a little

❑ To some extent

❑ Rather much

❑ Very much

Do you believe that stress is a contributing factor to your health problem today?

❑ No

- Yes, contributes somewhat
- Yes, contributes a lot

❑ Don´t know
